# Children’s perspectives on sugary snacks through elicitation techniques – repertory grid and generative method

**DOI:** 10.3389/fpsyg.2025.1342127

**Published:** 2025-03-18

**Authors:** Linglin Liang, Yue Yu

**Affiliations:** School of Art and Design, Zhejiang Sci-Tech University, Hangzhou, China

**Keywords:** sugary snacks, children’s attitude, child-centered approach, repertory grid, generative methods

## Abstract

**Background:**

Sugary snacks are prevalent in children’s daily lives and may impact their diet positively or negatively, yet few studies explore children’s perceptions and attitudes of these foods from their daily experiences in China.

**Aims:**

This study aims to (i) assess children’s perceptions and attitudes of sugary snacks and (ii) compare two child-centered elicitation techniques—Repertory Grid Technique (RGT) and Generative Method (GM)—based on Personal Construct Theory (PCT).

**Methods:**

A qualitative study was conducted with 31 children (6–10 years old) in Hangzhou and Fuzhou, China. Children participated in RGT (dichotomous comparisons using product cards) and GM (creative expression through drawing/clay) in one-on-one sections. The output of the task was analyzed for thematic analysis and descriptive statistics.

**Results:**

Themes identified included sensory, packaging, interaction, emotion, cognition, and socio-culture. Children expressed concern about food composition and showed limited understanding of sugar’s functions and cultural significance. The RGT elicited more product attributes (358 vs. 190 in GM), with a significant difference (*p* < 0.001). RGT generated a balanced mix of concrete and abstract attributes, while GM elicited more abstract attributes.

**Conclusion:**

The findings highlight that children can be educated about the formulation and cultural aspects of sugary snacks. Encouraging richer “in-mouth” and “in-body” interactive education could be beneficial. Adopting a child-centered approach fosters engaging conversations. RGT efficiently inspires children to generate both concrete and abstract product attributes and is easy to understand but less enjoyable. On the other hand, GM tends to generate more abstract and novel ideas that strongly reflect children’s preferences. However, it requires more time and cognitive effort to understand, though it maintains a higher level of enjoyment and engagement. Understanding these findings aids in developing nutrition education that captivates and engages children. Insights into elicitation techniques can guide researchers seeking to understand children’s perspectives effectively.

## 1 Introduction

Sugary snacks play a prominent role in children’s daily routines and are significantly shaped by social and cultural influences, offering not only energy but also a source of enjoyment. However, their excessive consumption has been linked to obesity and heightened cardiometabolic risks (World Health Organization, 2010; Zhang and Ma, 2017; Vos et al., 2017). Given that dietary preferences formed during childhood often persist into adolescence (Kelder et al., 1994), early nutrition education is essential to foster lifelong healthy eating habits.

Traditional nutrition education predominantly stems from parental perspectives (Matheson et al., 2002), overlooking children’s own views. However, understanding children’s perceptions of sugary snacks is imperative for designing engaging and effective programs which align with their interests and needs. Prior research has primarily delved into health perceptions, consumption factors (Battram et al., 2016; Roesler et al., 2021), and the influence of advertising on sugary beverages (Gesualdo and Yanovitzky, 2019; Russell et al., 2019). Little is known about how children personally interpret sugary snacks. Additionally, as children represent the forthcoming generation of eaters, understanding the types of interactions related to sugary snacks that children actively choose and relish can inspire innovative ideas for future food experiences (Willett et al., 2019; Højlund et al., 2020).

Personal construct theory posits that personal explanatory systems are shaped by past experiences, influencing subjective values, judgments, and future expectations. This process is dynamic and transcends age boundaries (Fransella, 2005; Kelly, 2005). Consequently, it is reasonable to assume that children formulate subjective interpretations of Sugary snacks based on their daily dietary encounters, constructing pre-existing cognitive knowledge that in turn affects their dietary behaviors and preferences.

In this study, we delve into the personal interpretations of Sugary snacks from the point of children based on their daily dietary experiences. The existing literature indicates two primary research avenues: elicitation and elaboration. Elicitation techniques serve the purpose of unveiling personal meanings, while elaboration techniques serve to enrich the framework of these personal meanings, complementing the elicitation process. The choice of elicitation techniques can yield attribute information that varies in significance and personal relevance. Drawing upon the means-end chain theory (Gutman, 1982), we understand that abstract attributes connected to emotions and experiences hold greater sway in consumer decision-making compared to tangible, concrete attributes. Hence, it is pertinent to compare the abstraction levels of attributes derived from diverse methodologies. Equally important is the volume of information garnered during elicitation interviews. A technique that elicits a greater number of attributes may be deemed more efficient (Breivik and Supphellen, 2003). Moreover, when designing programs involving children, it is vital to tailor tasks and settings to suit the skills and interests of the specific age group. A thoughtfully crafted, inspirational program has the potential to stimulate children’s expression, enhance their involvement, and elevate their enjoyment of the tasks (Galler et al., 2022).

Elicitation techniques can be categorized into two main types: comparative elicitation and non-comparative elicitation techniques, depending on their applications. One widely utilized comparative elicitation technique is the Repertory Grid Technique (RGT). RGT is particularly effective at exploring subjective experiences from a child’s perspective, all while minimizing researcher bias (Alexander and van Loggerenberg, 2005; Fransella and Neimeyer, 2005). Previous research suggests that RGT tends to capture concrete, directly perceivable attributes, while fewer statements are about abstract attributes, such as software and user experience, that cannot be directly observed (Süner and Erbuğ, 2016). In contrast, Generative Methods (GM), a non-comparative elicitation technique, has gained popularity in recent years for studies involving children. GM involves the creation of user-generated artifacts such as paintings and sculptures to uncover underlying needs and expectations (Sanders, 2002; Butler et al., 2007). Through GM, children may express themselves more freely compared to RGT, resulting in a more comprehensive personal interpretation. Generative artifacts can effectively concretize their ideas, often coupled with a conversation to facilitate a transitional space (Butler et al., 2007).

This exploratory study aimed to:

- Assess individual perceptions of Sugary snacks through child-centered elicitation techniques, with the goal of informing the development of food education programs and future engaging food experiences for children.
- Compare two elicitation techniques, the Repertory Grid Technique (RGT) and Generative Methods (GM), using consistent outcome measurements (quantity of elicited attributes, level of attribute abstraction, and participant ratings of the procedures).

## 2 Materials and methods

### 2.1 Participants and procedure

The objective of this study was to delve into children’s personal interpretations of Sugary snacks as part of research pertaining to educational toys. We chose to work with children aged 6–10 years, encompassing Primary 1–4, owing to their ample reservoir of experiences and knowledge in comparison to younger children. Additionally, this age group demonstrates a transition from family-dictated food choices to more autonomous decision-making in their dietary preferences (Hill, 2002; Zeinstra et al., 2007; Warren et al., 2008).

Given the inherently subjective nature of children’s interpretations of their personal experiences, each participant was subjected to two distinct elicitation techniques: the Repertory Grid Technique (RGT) and Generative Methods (GM). These techniques were administered separately, with a minimum interval of one week between sessions.

The effectiveness of the inspiration process hinged significantly on the participants’ grasp of the procedure and their overall experience during engagement (Breivik and Supphellen, 2003). To gauge this, participants were tasked with completing a questionnaire at the conclusion of the study. This questionnaire was adapted from the Smiley Face Likert Scale (SFL) and incorporated five smiley face images. The SFL encompassed ratings pertaining to participants’ comprehension of the task procedure, ease of manipulation, immersion in the task, and level of enjoyment. This tool proved to be instrumental in aiding children’s comprehension of numerical rating scales (Mazzone et al., 2008; Zaman et al., 2013). Notably, research suggests that children tend to provide positive ratings more frequently than negative ones. Therefore, employing a happy-to-happy scale, featuring five images of smiley faces with varying degrees of happiness, was deemed more appropriate (Hall et al., 2016) (the specific form of the SFLs can be found in the Supplementary Figure S1).

We recruited 31 children (14 males and 17 females) within the target age group through the Social Innovation and Sustainable Design Laboratory at Zhejiang Sci-Tech University, China. The average age of the participants was 8.3 years (±1.35). The sample predominantly came from the urban areas of Hangzhou and Fuzhou, two highly urbanized cities located in the southeastern coastal region of China. The participants were primary school students, all receiving China’s nine-year compulsory education. Most parents had a relatively high level of education, with at least a bachelor’s degree, which may have influenced their awareness of children’s food choices and healthy eating habits.

This study was granted exemption by the ethics committee of Zhejiang Sci-Tech University, due to the evaluation tests performed with subjects simply being focused on attitudes and experience, and no intrusive tests were performed that represent any danger to human health. Children and their parents were provided with an informational flyer detailing the research program and a consent form, which they were required to complete. We obtained consent for audio and picture recording. All participating children were explicitly informed that their involvement in these activities was part of a dissertation research project, and they had the option to decline participation without any repercussions. As a gesture of appreciation, each child received a small prize at the conclusion of the study to express our gratitude for their participation.

#### 2.1.1 Repertory grid technique

The selection of elements (products) for the RGT program is very important since the entire elicitation process hinges on their comparison. These elements should encompass a broad spectrum of subject matter while remaining relevant to the study’s scope (Fransella, 2003). To ensure this, we conducted a preliminary survey by distributing questionnaires before starting the study through an online platform. The goal was to investigate the types of sugary foods that children in the target age group are regularly exposed to, as well as the frequency of their exposure. A total of 126 questionnaires were received, with 124 valid responses. Parents were asked to rank the sugary foods their children encounter most frequently, from highest to lowest. Based on the data from the valid questionnaires, we calculated the high-frequency exposure rate for each sugary food (i.e., the percentage of times a food was ranked first by parents). The results showed that soft candies, cakes, and yogurt products had high-frequency exposure rates of 24.19, 22.58, and 20.97%, respectively, identifying them as the most encountered sugary foods among children. In addition, we selected three other foods with relatively high exposure rates—hard candies (13.71%), chocolate (8.87%), and ice cream (7.26%)—to include these six sugary snacks as elements in the RGT program. In our exploration of product attributes, visual representations played a pivotal role in clarifying the perceptual experience and providing tangible objects for manipulation (Töre Yargın, 2012; Kuru and Erbuğ, 2013). Consequently, we utilized product image cards as components within the elicitation program, supplemented by the provision of physical products procured from local supermarkets.

The researchers introduced the study using a scenario in which a supermarket is planning to launch new products and seeks children’s input as “sweet food critics” to provide feedback on existing products. Six types of sugary foods were presented as inspiration elements for the task. To streamline the study and facilitate its execution, children were given the opportunity to choose their two most preferred and two least preferred items from a selection of six categories of Sugary snacks as the focus of the interview. The configuration of these elements was randomized while ensuring diversity within the chosen items. While the interviews were rooted in the RGT program, we considered recommendations from relevant studies and tailored our approach to be more child-friendly. Specifically, we adopted a binary elicitation rather than a ternary one, as suggested by studies such as Bell and Butler (Bell et al., 2004; Butler et al., 2007). The researchers presented the four selected categories of Sugary snacks to the children in pairs, prompting discussions about their similarities and differences. Additionally, we incorporated a laddered interview process, consistently employing the question “why” to elicit implicit reasons. The research process was overseen by one primary researcher with the assistance of two colleagues. Upon completion of the study, questionnaires were distributed to gather participant feedback. The entire study process was documented through filming and subsequently transcribed for analysis.

#### 2.1.2 Generative methods

The Generative Methods (GM) approach allows children the flexibility to choose from various techniques for expressing their ideas, including drawing, clay modeling, collage, and even body language. Before commencing, we introduced each technique to ensure that children could independently utilize them. This preparedness was verified based on responses from parent questionnaires, and it was confirmed that each participant was already acquainted with these techniques as they were included in their respective preschool curricula. The procedure was introduced using a contextual story similar to that of the RGT, where a supermarket aims to launch new products and invites children to act as “sweet inventors” to create new types of sweets. The materials and methods for each generation technique were explained. Children were invited to choose one or more techniques to express their ideas, with assistance provided if they encountered difficulties in generating ideas. After the creation process, the children were interviewed about their products, explaining the meaning behind their creations. Follow-up questions such as “why” were used to uncover any underlying reasons behind their choices.

Throughout the study, three researchers were engaged in distinct roles. One researcher introduced the generative themes, another conducted interviews with the participants to capture generative artifacts, and a third researcher provided support and took notes. Upon concluding the study, questionnaires were disseminated to collect feedback from the participants. The children’s generative artifacts were meticulously photographed and recorded, while the interviews were audio-recorded and subsequently transcribed for in-depth analysis.

### 2.2 Data analysis

#### 2.2.1 Children’s personal interpretations of sugary snacks

**Figure 1:**
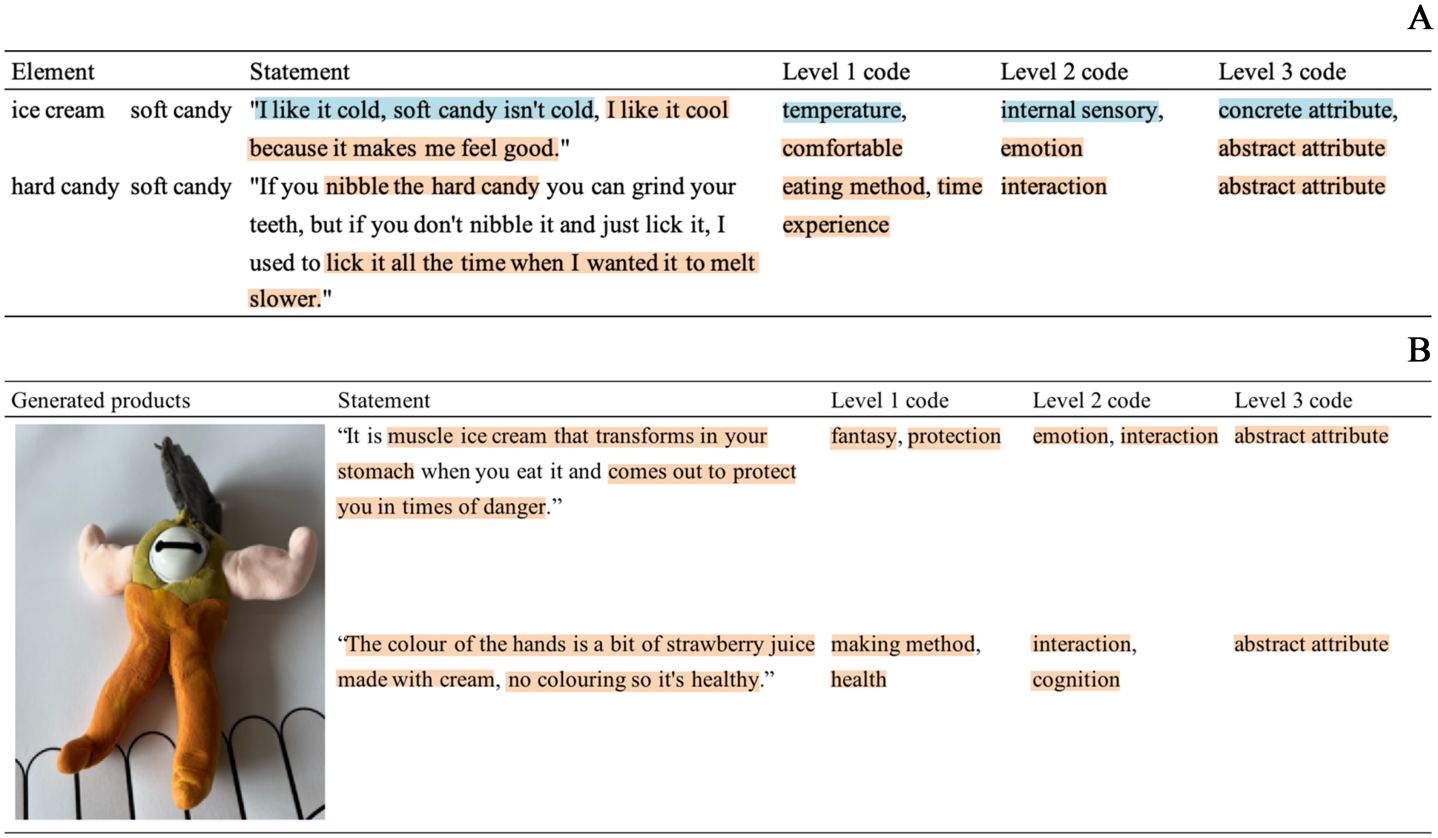
**(A)** RGT results coding process. **(B)** GM results coding process.

We analyzed the outcomes of each task, including transcripts (RGT and GM), as well as the generated artifacts such as drawings, clay models, and collages, both qualitatively and, when applicable, quantitatively. To align with the study’s objectives, we employed an inductive thematic analysis approach to qualitatively assess children’s perceptions and experiences of Sugary snacks. This bottom-up method involves identifying themes from text or images without pre-existing theories (Clarke, 2013), which aligns well with the exploratory nature of our study. All textual content and images were imported into NVIVO 7.1 to maintain privacy, with participant names replaced by numerical identifiers. The coding process, as depicted in Figure 1, followed these steps:

- Recognition and semantic classification of original utterances, providing information on product attributes perceived by children (Breivik and Supphellen, 2003). For example, in one child’s comparison between ice cream and yogurt, terms like “cold” and “liquid” were preliminarily coded as “temperature” and “state.”
- Identifying and coding additional core categories, drawing upon Gayler’s categorization of eating experiences in Human-food-technology Interaction (HFI) studies (Gayler et al., 2022). For example, the initial codes were eventually grouped into broader themes, such as “internal sensory,” which encompassed children’s descriptions of taste, texture, and temperature. During the process of defining these themes, special attention was given to the depth of interpretation and the context of the codes. For instance, in the theme “interaction,” we included not only descriptions of how the food was consumed but also behaviors mentioned by the children related to interacting with the food, such as how it was made or playful behaviors involving the food.

Each code was reviewed by a user experience researcher and a nutrition expert, and the core categories were discussed and finalized collaboratively. Additionally, we engaged with the participants to verify whether the thematic classifications accurately reflected their expressed content and experiences, as a lack of participant validation can lead to adult-centered bias (Petr, 1992). In the end, six core categories were defined: sensory, packaging, interaction, emotion, cognition, and socio-cultural. The detailed explanations of these dimensions are provided in Table 1.

**Table 1:** Explanation of core category codes.

Core category codes	Explanation	The level of abstraction of the attribute.
Sensory	It can be experienced from the body’s external senses (smell, sound, sight, and touch), and internal senses (taste, texture, temperature, digestion, and metabolism).	Concrete attribute
Packaging	Perceptible product appearance that is easy to handle and carry.	Concrete attribute
Interaction	Focuses on the interactive behaviors of people and food, including how food is used, formal or informal dining habits, and playful eating experiences.	Abstract attribute
Emotion	Emotional experiences triggered by food or interactive behaviors associated with it.	Abstract attribute
Cognition	Includes food-centered data, but not information that can be directly perceived. Examples include the source of the food, its composition, nutritional information, etc., or task-irrelevant values. Often depends on the participant’s personal background, values, aspirations, etc.	Abstract attribute
Socio-culture	Use food to capture and communicate personal or collective stories. Use eating experiences to make social connections. The impact of social relationships on eating habits, etc.	Abstract attribute

#### 2.2.2 Evaluation of RGT and GM setting

The second research objective involves comparing two distinct elicitation techniques along several dimensions:

- Amount of information: We assessed the quantity of information gathered by these techniques. Descriptive statistics were conducted on the survey data using SPSS 26.0, which included counting the number of attributes generated by both elicitation techniques. We further evaluated the impact of these techniques on the number of attributes generated through a paired sample t-test.
- Level of abstraction and personal interpretation: According to the Means-End Chain Theory (Gutman, 1982), each core category was subcategorized into concrete and abstract attributes, as outlined in Table 1. In the context of products, attributes fall into two categories: concrete and abstract. Concrete attributes are tangible qualities like color, size, or packaging, while abstract attributes encompass intangible aspects that evoke memories or personal significance due to their representation of symbolic values or cultural identity. In consumer research, abstract attributes are often more meaningful and can better reveal children’s attitudes and values.
- Participant evaluation: We statistically analyzed the retrieved Smiley Face Likert Scales (SFL) using SPSS 26.0, assigning coded values ranging from 1 to 5 based on the meaning associated with each smiley face. We calculated the mean and standard deviation of the rating questions. Additionally, researcher observations and verbal feedback from the participants were considered to further explore children’s overall experience with the two techniques from a qualitative perspective.

## 3 Results

### 3.1 Quantity of attributes and level of abstraction

In total, we obtained 358 personal concepts through the Repertory Grid Technique (RGT) and 190 personal concepts through Generative Methods (GM). The RGT method proved more effective in eliciting attributes from children, with respondents generating an average of 11.55 attributes compared to 6.13 attributes generated using the GM method. This difference between the two methods follows a normal distribution and was statistically significant (*p* < 0.001) as determined by a paired-sample *t*-test.

Additionally, the results from the RGT method showed a nearly equal distribution of concrete attributes (50.84%) and abstract attributes (49.16%). In contrast, the GM leaned toward eliciting more abstract attributes (59.47%).

### 3.2 Children’s personal interpretations of sugary snacks

#### 3.2.1 Repertory grid technique

The RGT program inspired children to generate a total of 358 personal concepts. Through semantic classification, 28 distinct attributes were identified, which were further grouped into core categories. Table 2 presents the core categories identified through content analysis, specific examples of attribute descriptions, and the number of attributes summarized within each category. The frequency of each core category highlights the key areas of focus for children when evaluating sugary foods. The analysis of qualitative data provides contextual information that helps explain the appeal of different attributes to children and reveals potential motivations behind their consumption behavior.

**Table 2:** Core attribute categories, example quotes, and number of attributes generated by the Repertory Grid Technique (*n* = 358).

Core attribute categories			Example quotes	Number of attributes
Concrete attribute	External sensory	Texture	“The cake is soft and fluffy, and the chocolate is crunchy.” “The chocolate is chewy and hard.”	101
		Shape	“The soft candies come in all kinds of shapes, and the chocolate is in square pieces.”	
	Internal sensory	Taste	“They all have lots of different flavors, like strawberry hard candy and strawberry yogurt.” “Some chocolate is bitter, I do not like to eat.”	69
		Temperature	“I like it cold, soft candy is not cold, I like it cool because it makes me feel good.”	
	Packaging	Easy to eat	“The hard candy has a stick to hold, but the chocolate does not have a stick and can make your hands messy.” “I like things I can lick right away or hold and eat, it’s very easy.”	12
Abstract attribute	Cognition	Health & Nutrition	‘Because it has milk inside, it can help me grow taller.’ ‘I do not like eating things that are too sweet, because it can make my teeth go bad easily.’	66
		Ingredient	“There are lots of food coloring.” “There should be additives in them. The cake cream has additives, the candy has additives, and the drinks have them too.”	
	Interaction	Eating method	“If you nibble the hard candy, you can grind your teeth, I used to lick it all the time when I want it to melt slower.”	56
		Making method	“Yogurt is fermented.” “There are many ways to use chocolate to make lots of desserts.”	
	Emotion	Comfortable & Happy & Refreshing	“Because I think the cold feeling is very nice.” “I like drinking the kind that flows and feels refreshing.”	33
	Socio-culture	Context	“Yogurt is usually eaten after meals, and cake is usually for birthdays.”	21

Children’s focus on external sensory attributes was the most prominent. Specifically, they were highly sensitive to the texture (soft/hard), shape, color, and state (solid/liquid/sticky) of the products. For instance, many children described the “*softness*” of soft candies and the “*hardness*” of hard candies, directly linking these sensory experiences to their preferences. Color and appearance were also frequently mentioned, with children noticing the variety of colors and shapes. However, these attributes were not direct factors in their decision to consume sugary foods but were instead associated with other aspects, such as health and flavor. Internal sensory attributes primarily involved perceived taste and temperature, with fewer mentions of a food’s smell. Children also discussed the product’s container and packaging, particularly in terms of convenience. For example, some children noted that the design of ice cream cones and lollipops made these foods easier to carry and eat, which increased their preference for them.

Regarding the abstract attributes, children showed significant concern about the health aspects of food. Many children mentioned the negative effects of food coloring and additives on health, with comments like “*food coloring is bad for your body.*” However, some children also recognized the positive role of food coloring in enhancing the appearance of food, such as “*food coloring makes candy look prettier.*” The interaction attributes highlighted children’s interest in the eating experience, particularly how different ways of consuming sweets influenced their perception of time and sense of control. For example, one child explained, *““I used to lick it all the time when I want it to melt slower.”* Emotional attributes were closely tied to the sensory characteristics of the food. For instance, some children mentioned that the “*cool*” and “*comfortable*” feeling from ice cream made them happy, while many others remarked that most sugary foods were *“too sweet,”* which made them feel “*sick*” or *“nauseated.”* These emotional responses demonstrate how sensory properties can trigger both positive and negative emotional experiences. Additionally, children rarely mentioned the socio-cultural attributes of food, though some described specific eating occasions and parental restrictions, such as the tradition of eating cake at birthday parties.

#### 3.2.2 Generative methods

Each of the 31 participants contributed at least one creative idea for a sugar-sweetened food, which they presented using various methods. They provided either written or verbal explanations of their products, with most children opting to craft three-dimensional models using clay. Some combined techniques, including drawing and clay modeling. After analyzing the participants’ creative work and descriptions, we identified 190 personal concepts and 26 attributes, which were then organized into core categories. Table 3 presents the attribute categories identified through content analysis, along with specific examples of generated products and their descriptions, as well as the number of attributes summarized within each category.

**Table 3:** Core attribute categories, example quotes, and number of attributes generated by the generative methods (*n* = 190).

Core attribute categories			Example quotes	Number of attributes
Concrete attribute	External sensory	Shape	“The hard candy is made in the shape of ice cream.” “It’s a lollipop shaped like an ice cream cone.” 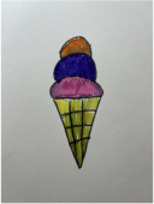	39
		Color	“There are many different colors, and they look really pretty.”	
	Internal sensory	Taste	“The different colors are different fruit flavors, like strawberry, grape, and mango. When the different flavors mix together, they taste really good.” “There’s white cream on top. Because the chocolate is bitter and the cream is sweet, when you take a bite, you get both bitter and sweet together.”	34
		Mixed taste experience		
	Packaging	Container	“The cupcake has pretty decorations on it.” 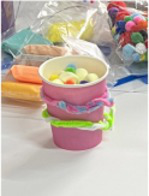	4
Abstract attribute	Cognition	Ingredient	“The added white sugar only has 40% sugar, so it’s not too sweet. That way, your teeth will not get cavities so quickly.” “My sweets do not have any additives. The cream is made from milk and yogurt, all natural with no food coloring. It’s made with eco-friendly ingredients.”	50
		Health & Nutrition	“My sweets can help with digestion.” “The colorful fluffy little balls are nutritious pellets that can help you grow taller.”	
		Structure	“The outer layer wraps around the filling inside the small ball. It’s a juicy gummy with a coating, and when you bite it, liquid comes out.”	
	Interaction	Game behavior	“My sweet treat is a chess set. The chessboard is made of candy wrappers, and the chess pieces are chocolate. You can play chess and eat at the same time. You can tear off a piece of the board and wrap up the chess pieces.” 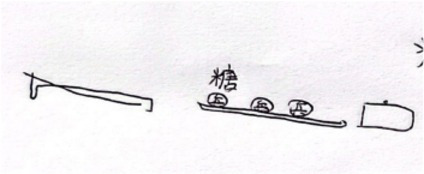	26
		Eating method	“On the outside, it’s marshmallow. First, you put it in your mouth and let the outer layer melt, then you chew the gummy inside.”	
	Emotion	Novelty	“It looks like a clown, very strange.”	30
		Fantasy & Fun	“It is muscle ice cream transforms in your stomach when you eat it and comes out to protect you in times of danger.” 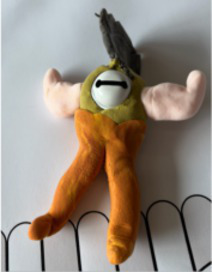	
	Socio-culture	Context	“This is an ice cream lollipop. When you want to eat ice cream in the winter, you can buy one.”	7

When creating their own sweets, children tended to describe their subjective perceptions of the sweets, highlighting a focus on health and ingredients. Unlike the results generated through the RGT method, children typically only described positive effects, often replacing unhealthy components in sugary foods with ingredients they perceived as healthier. For example, one child designed a candy made entirely from natural ingredients and explained, *“Because it’s made from pure fruit juice, there’s no food coloring, so it’s good for the body.”* Another child mentioned using xylitol instead of sugar, considering this choice *“very healthy,”* and emphasized that their sweet not only tasted good but also *“helps me grow taller.”* This demonstrates that children associate their preferred food ingredients with natural and health benefits.

The visual aspect of external sensory experience also played a significant role in children’s creations. The shapes of the sweets were often linked to fun themes, such as animals, monsters, and other fantasy characters, or were designed to resemble other foods (e.g., hard candies shaped like chicken legs or ice cream), evoking feelings of novelty, surprise, and amusement. Internal sensory experiences were also important, with many children exploring different flavor combinations and expressing the *“fun”* of mixing tastes to create a rich sensory experience. For example, some children enjoyed blending their favorite flavors together to enhance the taste, as one child explained: *“I like mixing different colored candies together so I can taste all the flavors at once.”* Another example involved combining different textures, such as wrapping one type of food with another: *“I put soft candy inside cotton candy, so when you bite into it, it melts but still has a chewy texture”*.

Interaction with food was also evident in children’s descriptions of their creative products, particularly in terms of playfulness and imaginative functions. For example, one child combined chocolate with chess, designing a chocolate chessboard and pieces that could be eaten while playing. Another child designed a candy with a *“protective”* function, imagining that the food would transform into battle guards inside the digestive system. Like the results from the RGT method, children made fewer references to the socio-cultural aspects of sweets.

### 3.3 Participant evaluation

Table 4 presents the questionnaire evaluation results based on participants’ assessments of the two programs. Quantitative analysis shows that participants generally found the RGT task easier to understand (*f* = 7.547, *p* < 0.05). Many younger children still expressed difficulty in understanding how to design and create a sweet during the GM task. However, when comparing the overall difficulty of the tasks, there was no significant difference between RGT and GM (*f* = 2.343, *p* > 0.05), with average ratings of 3.52 and 3.00, respectively.

**Table 4:** Mean and standard deviation of participant questionnaire (*n* = 31) score.

Evaluation dimension	Elicitation techniques	Mean ± SD	*f*	*p*
Task comprehension	RGT	3.71 ± 1.31	7.65	0.008
	GM	2.90 ± 1.66		
Task ease	RGT	3.52 ± 1.21	2.34	0.131
	GM	3.00 ± 1.44		
Task congruity	RGT	3.90 ± 1.06	0.01	0.916
	GM	3.87 ± 1.28		
Task fun	RGT	3.10 ± 1.42	13.11	0.001
	GM	4.29 ± 1.16		

To better understand these results, we combined participants’ verbal feedback with observations made by the researchers. Many children noted that in the RGT program, it was easier for them to identify the differences or similarities between two elements, largely because the task had a clearer structure. However, in the GM program, creating an entirely new sweet and describing its characteristics was considered more complex. Some children mentioned that during the creation process, they often chose to replicate craft projects they had made during extracurricular activities, or selected certain types of food because they were *“easier to draw.”* For example, one child said, *“I drew an ice cream with lots of colors because I have drawn it before”*.

The researchers’ observations further supported this feedback. We found that some children struggled to describe their creations in detail, lacking specific explanations for their creative ideas. During the GM task, some children provided vague verbal descriptions of their creations, unable to clearly convey their ideas. For instance, one child created a candy model but described its taste and texture only as *“sweet”* or *“soft”* without offering further details. However, there were also children who demonstrated clear and creative thinking. These participants not only created unique sweets but also provided detailed explanations of the ingredients and design intentions. For example, one child made a *“pencil candy”* and thoroughly explained how the different colors represented various flavors and sensory experiences, along with how and when it should be eaten.

In terms of task congruity, there was no significant difference between the two techniques (*f* = 0.011, *p* > 0.05), suggesting that both programs were generally consistent with children’s ways of thinking. However, in terms of enjoyment, a significant difference was found between the two techniques (*f* = 13.105, *p* < 0.05), with children generally finding the GM method to be more enjoyable than the RGT method. Some children commented that the questioning style in the RGT task made them feel like they were taking a *“test,”* where they were searching for a correct answer rather than responding based on their own experiences. One child mentioned, *“I felt like these questions were too easy, and I could not get them wrong.”* In contrast, the GM method was perceived as more creative and offered a greater sense of freedom. Children were more likely to view it as a game or a process of creative expression, which allowed them to confidently express their preferences, emotions, and imagination.

## 4 Discussion

This study delves into children’s individual interpretations of Sugary snacks encompassing sensory, packaging, interaction, emotion, cognition, and socio-cultural dimensions using child-centered elicitation techniques. In the subsequent sections, we will delve into the outcomes of two elicitation methods, their potential contributions to the design of impactful nutritional education programs and child-friendly food products, as well as innovative concepts for future food interactions. We will also scrutinize the efficacy of these two elicitation techniques, the level of abstraction characterizing the attributes they elicit, and the feedback from participants. Furthermore, we will assess the strengths and limitations of both approaches.

### 4.1 Sugary snacks based on a child’s perspective

From our elicitation results, it becomes evident that children possess a keen awareness of the ingredients and adverse health consequences associated with Sugary snacks, even when the conversation does not explicitly steer toward health aspects. Their awareness primarily revolves around their perceptions of high sugar content and additives in these products, along with the health risks associated with excessive sugar consumption, such as tooth decay and obesity. These findings align with previous studies, which highlighted that children often associate high sugar content with unhealthy beverages (Bucher and Siegrist, 2015). One plausible reason for this heightened awareness might be the integration of various food education activities into the recommended curriculum by the Chinese Ministry of Education since 2017. These activities encompass not only conventional classroom teaching but also engaging and interactive learning approaches, such as food education workshops, service system design, and children’s literature (Zhang, 2018; Chen, 2018; Zhou and Zhang, n.d.). As early as grades 1 and 2, children start grasping the nutritional value of various foods, the principles of healthy eating, and the scientific rationale behind dietary choices (Zhang et al., 2020).

Conversely, children exhibit significantly less awareness of the functional and cultural aspects of sugar, such as its potential to provide timely energy and enhance metabolism when consumed in moderation. Even if they are cognizant of some of these functions of Sugary snacks, they tend to attribute them to sensory pleasure, emotional satisfaction, and aesthetic experiences rather than recognizing them as effects of sugar. Interestingly, in the GM results, some children suggested using alternative ingredients with no adverse health effects to achieve sweetness in desserts, while still satisfying their preference for sweet flavors. This aligns with the global trend of sugar reduction programs aiming to influence the availability of sugary products through reformulation (Public Health England, 2017). Although functional sugars and low-calorie sweeteners (LCS) have gained popularity in China’s food industry, consumers are increasingly seeking more “natural” alternatives to traditional LCS. They desire foods that are both sweet and delicious (Stanner and Spiro, 2020). Given that the upcoming generation will play a pivotal role in food production and preparation scenarios (Willett et al., 2019; Højlund et al., 2020), there is considerable value in incorporating the promotion of functional sugar sources and encouraging children to advocate for reformulating currently sugary foods into children’s food education programs.

Regarding interactive experiences with Sugary snacks, our findings suggest an opportunity to develop richer “in-mouth” and “in-body” interactions. In the RGT study, we observed that children had diverse and detailed sensory experiences when consuming Sugary snacks. They paid close attention to factors like the texture and state of the food, and they noticed how different eating styles, such as chewing or savoring, influenced the flavor of these treats in their mouths. In the GM study, children expressed a desire for more intricate taste experiences, seeking a blend of flavors and textures that would evoke novel and surprising emotional responses. This discovery aligns with current research in the field of Human-Food-Technology Interaction (HFI), which is increasingly recognizing the significance of both external (sight and taste) and internal (digestive metabolism and related sensations) aspects of the eating experience. Previously, the focus was primarily on external sensations, with limited attention given to internal experiences, such as those arising from chewing or the digestive process (Gayler et al., 2022). The mouth plays a pivotal role in creating a multimodal sense of taste, integrating taste, smell, and touch (sensitivity to changes in texture, temperature, and state). It also contributes to the emotional aspects of the eating experience (Kerruish, 2019). Understanding that digestion commences in the mouth, children may not be fully aware of the sensations occurring within their bodies during consumption, but they can learn to recognize and comprehend them. For example, they can learn about the ingredients in yogurt that aid digestion or how consuming ice cream at certain temperatures can impact the digestive system negatively. Increasing this awareness has the potential to reshape their relationship with food and enhance their ability to regulate eating behaviors.

Interactive technologies, such as Augmented Reality (AR)-based body games that illustrate the digestive process and promote proper chewing techniques (Arza et al., 2018), offer promising avenues for enhancing “in-mouth” and “in-body” experiences. These technologies have the potential to educate and engage children in understanding the intricate processes occurring within their bodies during and after food consumption, fostering a positive and informed relationship with food.

### 4.2 Evaluation of RGT and GM setting

The utilization of two distinct elicitation techniques yielded contrasting outcomes. In contemporary child-centered research, there is a growing emphasis on capturing children’s perspectives. This necessitates the utilization of methods that are both straightforward and efficient in comprehending their ideas, visions, and aspirations. The following section provides a concise overview of the attributes of the two elicitation techniques, encompassing their efficiency, level of abstraction in elicited attributes, and procedural setup. This summary assists researchers in selecting and refining these techniques in alignment with the specific objectives of their studies.

The elicitation results demonstrate that the RGT program effectively stimulates children to generate a greater number of product-related attributes. Working with child participants revealed that having well-defined procedures in place facilitated their expression. Despite the presence of generative artifacts as a medium, employing a structured approach enhanced communication between children and researchers. This structured approach not only saved time but also streamlined the research process efficiently.

In contrast, the GM method yielded a higher percentage (59.47%) of abstract properties compared to the RGT. Previous research has shown that the RGT program tends to elicit concrete aspects of the discussed product (Bech-Larsen and Nielsen, 1999; Hassenzahl and Wessler, 2000). This tendency may stem from the typical basis of RGT programs, which rely on comparisons of existing products or prototypes. However, we found that using a stepwise questioning and ranking approach can further elicit children’s abstract experiential attributes related to interaction, emotions, and cognition with the product. In the RGT study, the ratio of abstract to concrete attributes generated by children was nearly equal, which reflects a child-driven pattern of attitudes and experiences related to the product. However, this method struggles to explore future or non-existent concepts and experiences. In contrast, the GM method elicited attributes at a higher level of abstraction, and abstract attributes are generally considered more important than concrete ones (Bech-Larsen and Nielsen, 1999). As a result, the creations roughly reflected children’s preferences, such as favorite flavors and shapes. Children also expressed more positive effects or emotional information, offering more imaginative or novel creative ideas.

Regarding the procedural dimension, the RGT program proved easy for children to comprehend and navigate. Children grasped the structured comparison procedure involving specific products and images efficiently. After a few product comparisons, they autonomously initiated subsequent comparisons and sorting. Even children under 10 years of age easily comprehended binary comparisons. Conversely, the GM method, despite the reference elements provided, posed some challenges for children in terms of directly creating new Sugary snacks. Some children required ample time to figure out the process. Structured processes, such as scaffolded drawings, aided children’s understanding and experimentation, fostering focused thinking (Wiggins et al., 2019). The RGT program’s repetitive comparison and sequencing process could lead to boredom and distraction once the initial curiosity waned. In contrast, the GM process was relatively enjoyable for children and promoted sustained concentration. Integrating elicitation methods into games enhance children’s comprehension of the task. Games, recognized as engaging tools, encouraged conversation and collaborative learning among players. This facilitated the exchange of diverse practices and viewpoints, exemplified by activities like storytelling using a story card game (Kjaersgaard et al., 2021).

In summary, our findings shed light on the distinctive qualities of employing the RGT and GM methods to capture children’s perspectives and experiences regarding Sugary snacks. The RGT approach proves to be efficient and user-friendly, featuring a well-structured process. It leverages tangible product comparisons to elicit concrete attributes derived from existing products, with the potential for incorporating elaboration techniques to evoke more abstract ideas. However, it may be uninteresting and boring. The GM method generated insights and ideas related to future preferences and more creative concepts. Although it required more time and cognitive effort, children were able to maintain their focus and enjoyment throughout the process.

### 4.3 Limitations and further research

While this study employed random sampling during data collection, it remains a cross-sectional study with certain limitations. Firstly, the social and cultural context, as well as the upbringing and educational environment of the children, can influence the study’s results. The sample in this study primarily consisted of elementary school students from Hangzhou and Fuzhou, China, all of whom are enrolled in the nine-year compulsory education system. While these regions have high levels of economic development and education quality, we did not systematically collect data on participants’ socioeconomic backgrounds, such as specific household income. Socioeconomic factors may impact children’s exposure to and attitudes toward sugary foods, particularly when comparing families with different income levels and educational attainment. These differences could be more pronounced. Therefore, the participants’ experiences and views may more closely reflect the social and cultural background of middle- and upper-income families in these areas, and may not be broadly generalizable, especially to other regions in China with significant socioeconomic differences. This limitation restricts the generalizability of our findings. Future research should aim to include a more diverse sample, particularly children from various socioeconomic backgrounds, to better understand how socioeconomic factors influence food preferences and health-related behaviors.

Furthermore, children’s verbalizations and proficiency with generative techniques can influence study results. Despite RGT and GM’s efforts to collect user-centric data compared to traditional research methods, the researcher’s interpretation of children’s language and artifacts still holds significance. To enhance future research, minimizing the subjective element in data interpretation is crucial for a more comprehensive analysis.

## 5 Conclusion

Research into children’s individual interpretations of Sugary snacks remains limited. This study addresses this gap by utilizing child-centered Repertory Grid Technique (RGT) and Generative Methods (GM) elicitation techniques to explore children’s concerns regarding these foods. We investigate the attributes that matter to children and how they envision positive interactions with sugar-sweetened products. Our findings highlight children’s concerns about the composition of these foods and their limited awareness of sugar’s functions and cultural significance. Additionally, children emphasize the importance of rich sensory experiences during consumption. This information offers valuable insights into the future of nutrition education, both in terms of content and delivery.

We also discuss the advantages and limitations of the two elicitation techniques. The RGT program is characterized by efficiency and ease of participation due to its structured process. It leverages specific product comparisons and elaborations to yield concrete and abstract attributes based on real products. However, it may lack the intrinsic interest of children. On the other hand, the GM method offers insights into future preferences and generates more creative ideas, though it requires more time and cognitive effort. Despite this, children were able to maintain focus and enjoyment. Future research should focus on assessing the efficacy of these elicitation results and further improving methods that are easy for children to understand, engaging, and efficient.

## Data Availability

The raw data supporting the conclusions of this article will be made available by the authors, without undue reservation.
