# Supplementary figures and images for: Children’s perspectives on sugary snacks through elicitation techniques – repertory grid and generative method
Source: Front Psychol. 2025 Mar 18;16:1342127. doi: 10.3389/fpsyg.2025.1342127 (PMC11959041; doi:10.3389/fpsyg.2025.1342127)

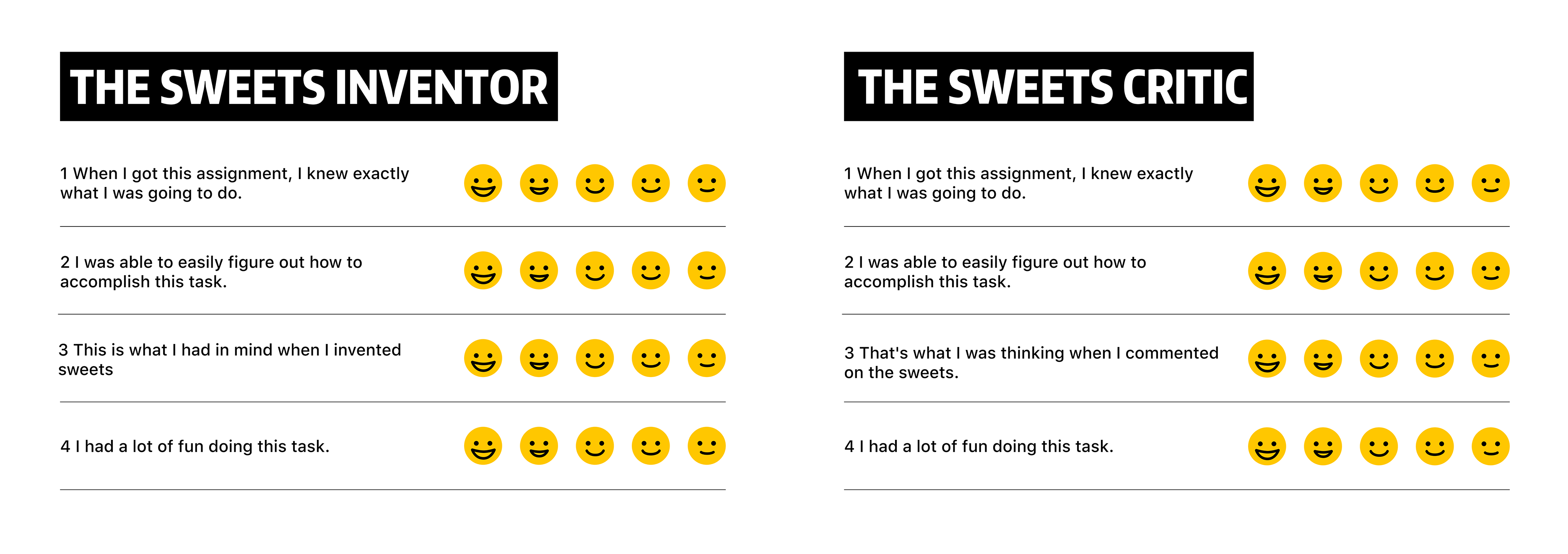

Supplement: Supplementary file 1 [file Image_1.tif]
